# Physical and psychological symptoms of quality of life in the CHART randomized trial in head and neck cancer: short-term and long-term patient reported symptoms

**DOI:** 10.1038/sj.bjc.6690829

**Published:** 1999-12

**Authors:** G O Griffiths, M K B Parmar, A J Bailey, on behalf of the C. H. A. R. T. Steering Committee

**Affiliations:** Cancer Division, Medical Research Council Clinical Trials Unit, 222 Euston Road, London NW1 2DA, UK

**Keywords:** symptoms, head and neck cancer, radiotherapy, CHART

## Abstract

The randomized multicentre trial of continuous hyperfractionated accelerated radiotherapy (CHART) versus conventional radiotherapy in patients with advanced head and neck cancer showed no good evidence of a difference in any of the major clinical outcomes of survival, freedom from metastases, loco-regional control and disease-free survival. Therefore an assessment of the effect of treatment on physical and psychological symptoms is vital to balance the costs and benefits of the two treatments. A total of 615 patients were asked to complete a Rotterdam Symptom Checklist and the Hospital Anxiety and Depression Scale, which cover a variety of physical and psychological symptoms, at a total of ten time points. The data consisted of short-term data (the initial 3 months) and long-term data (1 and 2 years). The short-term data was split into an exploratory data set and a confirmatory data set, and analysed using subject-specific and group-based methods. Differences were only claimed if hypotheses generated in the exploratory data set were confirmed in the confirmatory data set. The long-term data was not split into two data sets and was analysed using a group-based approach. There was evidence of significantly worse symptoms of pain at day 21 in those treated with CHART and significantly worse symptoms of cough and hoarseness at 6 weeks in those treated conventionally. There was also evidence to suggest a higher degree of decreased sexual interest at 1 year and sore muscles at 2 years in those treated with conventional radiotherapy. There is no clear indication that one regimen is superior to the other in terms of ‘quality of life’, generally the initially more severe reaction in the CHART group being offset by the longer duration of symptoms in the conventionally treated group. © 1999 Cancer Research Campaign


*British Journal of Cancer* (1999) **81**(7), 1196–1205
© 1999 Cancer Research Campaign
Article no. bjoc.1999.0829

### Physical and psychological symptoms of quality of life in the CHART randomized trial in head and neck cancer: short-term and long-term patient reported symptoms

**GO Griffiths, MKB Parmar and AJ Bailey on behalf of the CHART Steering Committee**

Cancer Division, Medical Research Council Clinical Trials Unit, 222 Euston Road, London NW1 2DA, UK

**Summary** The randomized multicentre trial of continuous hyperfractionated accelerated radiotherapy (CHART) versus conventional radiotherapy in patients with advanced head and neck cancer showed no good evidence of a difference in any of the major clinical outcomes of survival, freedom from metastases, loco-regional control and disease-free survival. Therefore an assessment of the effect of treatment on physical and psychological symptoms is vital to balance the costs and benefits of the two treatments. A total of 615 patients were asked to complete a Rotterdam Symptom Checklist and the Hospital Anxiety and Depression Scale, which cover a variety of physical and psychological symptoms, at a total of ten time points. The data consisted of short-term data (the initial 3 months) and long-term data (1 and 2 years). The short-term data was split into an exploratory data set and a confirmatory data set, and analysed using subject-specific and group-based methods. Differences were only claimed if hypotheses generated in the exploratory data set were confirmed in the confirmatory data set. The long-term data was not split into two data sets and was analysed using a group-based approach. There was evidence of significantly worse symptoms of pain at day 21 in those treated with CHART and significantly worse symptoms of cough and hoarseness at 6 weeks in those treated conventionally. There was also evidence to suggest a higher degree of decreased sexual interest at 1 year and sore muscles at 2 years in those treated with conventional radiotherapy. There is no clear indication that one regimen is superior to the other in terms of ‘quality of life’, generally the initially more severe reaction in the CHART group being offset by the longer duration of symptoms in the conventionally treated group. © 1999 Cancer Research Campaign

**Keywords:** symptoms; head and neck cancer; radiotherapy; CHART

In 1990 two parallel randomized controlled multicentre clinical trials were initiated to compare the effects of conventional radiotherapy, a daily dose given 5 days per week for 6–6.5 weeks, versus continuous hyperfractionated accelerated radiation therapy (CHART), given 3 times daily over 12 consecutive days (including weekends). The trials were conducted in patients with locally advanced non-small-cell lung cancer (NSCLC) and locally advanced head and neck cancer. Between 1 April 1990 and 31 March 1995, a total of 563 patients with NSCLC and 918 with head and neck cancer were entered by 13 centres. The results of these trials on the clinical endpoints have been published (Dische et al, 1997; Saunders et al, 1997).

Patients randomized on or after 1 October 1990 and before 1 December 1993 in the ten UK centres were asked to complete both a Rotterdam Symptom Checklist (De Haes et al, 1990) and a Hospital Anxiety and Depression Scale (Zigmond and Snaith, 1983) on a total of ten occasions, in order to assess quality of life (QOL) symptoms. The results of the analysis of the patient-completed QOL measurements for the NSCLC trial have been presented (Bailey et al, 1997) and published (Bailey et al, 1998).

The most recent published clinical results of the analysis for the head and neck trial found that there was no evidence of a difference between patients treated with CHART and conventional treatment in terms of any of the major clinical outcome measures of survival, freedom from metastases, locoregional control and disease-free interval (Dische et al, 1997). Given these results an important question remains: Is there any difference in the quality of life between the two treatments? In this paper we report the results of the comparison of CHART and conventionally treated patients in terms of patient-reported physical and psychological symptoms.

There are problems concerned with the analysis of QOL data due to its multidimensional and longitudinal nature and the possibility of informed censoring through missing information. Solutions have been suggested, but presently none satisfy all of these issues in combination. In this analysis the aim throughout has been to keep the methods of analysis simple (Cox et al, 1992) in order to minimize the number of assumptions made and to ensure that the presentation and interpretation of the results are as clear as possible. To do this each individual symptom was considered separately and the data were analysed separately for short-term (first 3 months) and long-term effects (1 year and 2 years). Such an approach also corresponds to clinically relevant times, representing periods of acute and late morbidity. For the short-term analysis a subject-specific approach and a group-based

*Received 2 November 1998*
*Revised 15 April 1999*
*Accepted 16 June 1999*

*Correspondence to:* GO Griffiths

Members of the CHART Steering Committee at the onset of the trial: A Barrett (Chairman), SJ Arnott, D Ash, CK Bomford, PJ Bourdillon, B Cottier, M Cuthbert, P Dawes, S Dische, W Forbes, A Harvey, JM Henk, TA Hince, AH Laing, RH MacDougall, DAL Morgan, FE Neal, H Newman, MKB Parmar, AG Robertson, RI Rothwell, MI Saunders, VH Svoboda, RP Symonds, JS Tobias, MJ Whipp, H Yosef.

approach were adopted to address the question of whether there was good evidence of a large difference between the two treatments over the first 3 months. To address the problem of multiple comparisons the short-term data were split into two data sets, one to generate hypotheses and one to prospectively test them. For the long-term analysis a group-based approach was adopted to address the question of whether there was good evidence of a large difference between the two treatments for those patients surviving to 1 year and those surviving to 2 years. It was not possible to consider longer term data as it was only collected for the first 30 months following start of radiotherapy. A subject-specific approach was not appropriate for the long-term data due to the small number of patients with information at these time points. It should be emphasized that the primary aim of the analysis was to assess the evidence for differences in reported symptoms between CHART and conventional radiotherapy, rather than to investigate variation over time in individual treatment groups.

### METHODS

The methods used in this analysis are described in detail in the NSCLC paper (Bailey et al, 1998) and therefore we only give them in summary in this paper.

### Assessment of symptoms

To assess symptoms of quality of life, the Rotterdam Symptom Checklist (RSCL) (De Haes et al, 1990) and the Hospital Anxiety and Depression Scale (HADS) (Zigmond and Snarth, 1983) were both used. The RSCL is a patient-completed questionnaire designed to be used in studies of cancer patients. It comprises 30 core symptoms covering two domains (physical and psychological), to which four symptoms relevant to this patient group were added (cough, coughing up blood, hoarseness and restlessness). In addition, the questions on low back pain and abdominal ache were replaced by pain. Patients recorded their overall experience of these 33 symptoms during the previous week using a 4-point categorical scale (0 = not at all, 1 = a little, 2 = moderately, 3 = very much). The HADS is a 14-item self-rating questionnaire, seven items concerning anxiety and seven depression, again using a 4-point categorical scale (0–3). The seven-item scores (0–3) of the anxiety questions were summed giving an overall score for anxiety of between 0 and 21; the same calculation was performed to obtain a score for depression. These scores were used to determine whether a patient, during the previous week, was considered normal (a score of 0–7), having borderline clinical anxiety or depression (a score of 8–10), or as a probable clinical case (a score of 11–21). In our analysis, patients only had a score for anxiety or depression at a particular assessment if they had responded to all seven relevant questions.

Patients were asked to complete these questionnaires before the start of treatment, at day 21, day 28, week 6 and at 3, 6, 12, 18, 24 and 30 months from the date of start of radiotherapy. These time points were selected to coincide with the collection of the clinical data and to assess patients when the side-effects of treatment were likely to be most severe. All data were collected by designated research nurses at each of the centres; each centre was visited to ensure that the research nurses and data managers were familiar with the procedures for data collection and handling of patient queries. Completed questionnaires were sent to the Medical Research Council Cancer Trials Office in Cambridge for checking and processing and were managed using the COMPACT program (COMPACT Steering Committee, 1996) and analysed using SAS (1989).

### Analysis

The data were analysed separately for short-term symptoms and long-term symptoms to circumvent the problem of missing data (Cox et al, 1992). The proportion of missing information was small during the first 3 months (short-term).

#### *Short-term*

The subject-specific approach is a method which considers the individual as the basic ‘unit’ of analysis. For each individual the severity of each symptom was plotted against the assessment time and then the area under the curve (AUC) was calculated. The total AUC score was standardized by dividing by the number of days between the first assessment and the final assessment, resulting in the standardized area under the curve (SAUC) being calculated for each patient, for each symptom. The SAUC can be interpreted as a type of weighted average of the responses over time for a symptom. The assumptions that were made in the AUC approach of analysis were as follows:

1. Patients who had data for a symptom missing consistently after a certain time point but for whom data were expected were not distinguished from patients who had died and thus for whom no data were expected;
2. Patients who had no data or only data at a single assessment for a symptom could not contribute to the analysis and were omitted;
3. Patients with missing data at the pretreatment assessment were excluded;
4. If a single data point was missing between two time points the missing value was imputed, a linear trend between time points was assumed. However, if two or more consecutive data points were missing for a particular symptom the patient was omitted from the analysis.
   The Mann–Whitney test (Altman, 1991) allowing for ties was used to formally test for a difference in SAUC scores between treatments for each symptom.

The group-based approach considers the proportion of all patients in each treatment group falling into each symptom category over time. This summary of the data gives an impression of the severity of each symptom at each specific time in the trial, and can be useful in highlighting changes in the distribution of patient response at particular times during follow-up. For each individual symptom the proportion of patients with moderate/severe values (or borderline/case for HADS) at each assessment were plotted and compared at each time point between treatments using a $\chi^2$ test.

To address the problem of multiple comparisons because of the many symptoms studied, we randomly split the short-term data into two subsets. This allowed hypotheses to be generated from the first data set ‘the exploratory data set’ and prospectively tested on the second data set ‘the confirmatory data set’. Differences were only claimed if they were ‘confirmed’ in this second data set; the reasoning for this split is discussed more fully in the NSCLC paper (Bailey et al, 1998). For the short-term group-based analysis the number of patients in both the exploratory and confirmatory data

© 1999 Cancer Research Campaign sets was sufficient to reliably detect a difference between treatments of 20% or more at any one time point (50% vs 70%, two-sided, power = 90%, α = 5%).

### Long-term

For the long-term symptom data at 1 year and 2 years, the analysis is performed conditionally on those patients who have survived to those time points and is therefore independent of any missing data (because of death) up to these time points. The number of patients with long-term data is diminished compared to those with short-term data and so the data were not split into an exploratory and confirmatory data set. Instead a group-based analysis was adopted for all patients with data at 1 and 2 years in order to investigate treatment differences for those patients surviving to these times. It should be noted that as a consequence there were no clear hypotheses to be tested and thus these analyses were largely exploratory in nature.

### RESULTS

### Patients

Of the 918 patients recruited into the trial, 615 were entered into the QOL study (373 CHART, 242 conventional radiotherapy). It should be noted that there was a 3:2 randomization in favour of CHART. Details of the patient characteristics for these 615 patients were broadly similar to the total data set of all 918 patients (Dische et al, 1997). Compliance was very good, with 87% of the total number of expected questionnaires received, and 78% 'fully' completed, disregarding the symptom decreased sexual interest.

### Pretreatment symptoms from the RSCL

Pretreatment data were available for 32 of the 33 items on the RSCL for at least 95% of patients (582/615), the exception being decreased sexual interest, where data were available for only 79% of patients (488/615). The frequency and severity of symptoms which were reported by patients before the start of treatment are shown in Figure 1, in decreasing order of prevalence. Using a Mann–Whitney test it was found that there was no good evidence that the distribution of severity was different in the two treatment groups.

### Pretreatment symptoms from the HADS

The percentage of patients with normal, borderline or case anxiety and depression at the pre-treatment assessment are given in Table 1. The proportion of patients with borderline or case anxiety and depression were similar in the two treatments. Differences between the sexes are discussed later.

**Table 1** Pretreatment HADS anxiety and depression scores, split by sex (all patients)

| | Overall (%) | Male (%) | Female (%) |
|---|---|---|---|
| *Anxiety* | | | |
| Normal (0–7) | 410 (71) | 326 (77) | 84 (56) |
| Borderline (8–10) | 87 (15) | 58 (14) | 29 (19) |
| Case (11–21) | 77 (13) | 41 (10) | 36 (24) |
| Patients with a score | 574 (93) | 425 (94) | 149 (92) |
| Total patients | 615 | 453 | 162 |
| *Depression* | | | |
| Normal (0–7) | 518 (89) | 391 (90) | 127 (85) |
| Borderline (8–10) | 33 (6) | 24 (6) | 9 (6) |
| Case (11–21) | 31 (5) | 18 (4) | 13 (9) |
| Patients with a score | 582 (95) | 433 (96) | 149 (92) |
| Total patients | 615 | 453 | 162 |

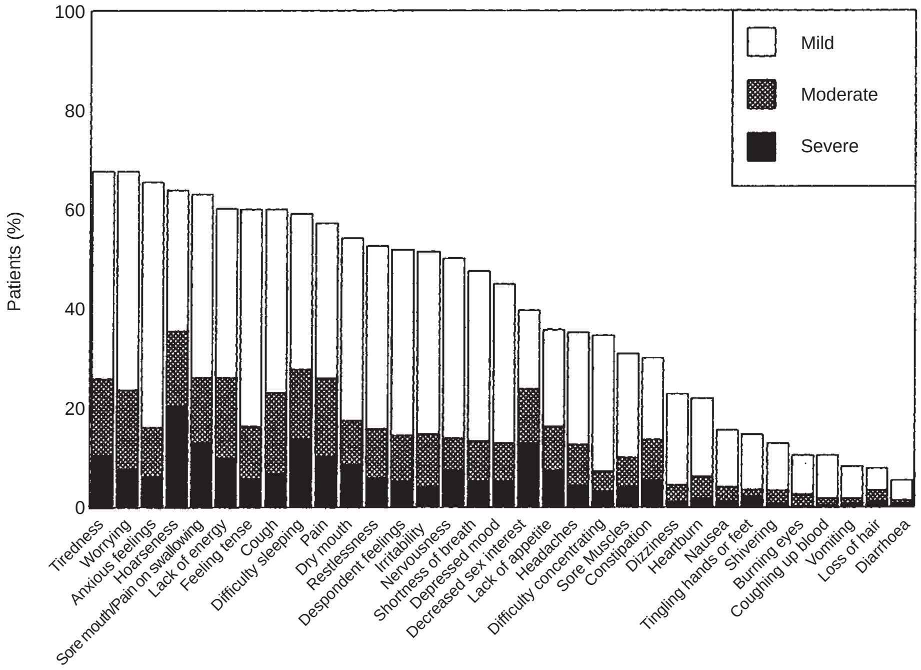

**Figure 1** Percentage of patients reporting symptoms from the RSCL before start of treatment (all patients)

© 1999 Cancer Research Campaign

**Table 2** Results of SAUC analysis for the RSCL symptoms (exploratory data set)

| Symptom | Median | | Interquartile range | | Difference between medians | Mann–Whitney *P*-value |
|---|---|---|---|---|---|---|
| | CHART | Conventional | CHART | Conventional | | |
| Constipation | 0.62 | 0.35 | 0.15–1.15 | 0–1.04 | 0.27 | 0.018[a] |
| Tiredness | 1.25 | 1.12 | 0.88–1.73 | 0.85–1.88 | 0.13 | 0.821 |
| Feeling tense | 0.58 | 0.46 | 0.12–1.00 | 0.12–1.00 | 0.12 | 0.456 |
| Nervousness | 0.38 | 0.27 | 0–0.88 | 0–1.00 | 0.11 | 0.322 |
| Lack of energy | 1.12 | 1.04 | 0.73–1.73 | 0.81–1.68 | 0.08 | 0.763 |
| Difficulty concentrating | 0.30 | 0.23 | 0–0.90 | 0–0.96 | 0.07 | 0.300 |
| Pain | 1.00 | 0.94 | 0.62–1.50 | 0.38–1.46 | 0.06 | 0.310 |
| Difficulty sleeping | 0.75 | 0.69 | 0.27–1.23 | 0.15–1.19 | 0.06 | 0.281 |
| Depressed mood | 0.50 | 0.46 | 0.12–1.00 | 0–1.00 | 0.04 | 0.411 |
| Sore mouth/pain on swallowing | 1.42 | 1.38 | 0.98–1.98 | 0.96–2.25 | 0.04 | 0.698 |
| Shortness of breath | 0.27 | 0.23 | 0–0.88 | 0–0.92 | 0.04 | 0.607 |
| Restlessness | 0.65 | 0.62 | 0.20–1.02 | 0.23–1.00 | 0.03 | 0.413 |
| Headaches | 0.15 | 0.15 | 0–0.54 | 0–0.62 | 0 | 0.707 |
| Worrying | 0.73 | 0.73 | 0.27–1.27 | 0.23–1.12 | 0 | 0.418 |
| Sore muscles | 0.27 | 0.27 | 0–0.75 | 0–0.65 | 0 | 0.296 |
| Vomiting | 0 | 0 | 0–0.33 | 0–0.35 | 0 | 0.160 |
| Dizziness | 0 | 0 | 0–0.33 | 0–0.35 | 0 | 0.831 |
| Diarrhoea | 0 | 0 | 0–0 | 0–0 | 0 | 0.555 |
| Tingling hands or feet | 0 | 0 | 0–0.26 | 0–0.35 | 0 | 0.505 |
| Loss of hair | 0 | 0 | 0–0.27 | 0–0.27 | 0 | 0.890 |
| Burning eyes | 0 | 0 | 0–0.15 | 0–0.17 | 0 | 0.724 |
| Coughing up blood | 0 | 0 | 0–0.27 | 0–0.23 | 0 | 0.419 |
| Shivering | 0 | 0 | 0–0.38 | 0–0.38 | 0 | 0.469 |
| Despondent feelings | 0.46 | 0.46 | 0–1.12 | 0–1.00 | 0 | 0.441 |
| Anxious feelings | 0.65 | 0.65 | 0.23–1.12 | 0.15–1.00 | 0 | 0.928 |
| Nausea | 0.15 | 0.19 | 0–0.54 | 0–0.69 | –0.04 | 0.664 |
| Irritability | 0.66 | 0.73 | 0.27–1.17 | 0.25–1.00 | –0.07 | 0.526 |
| Lack of appetite | 0.88 | 1.00 | 0.54–1.58 | 0.35–1.92 | –0.12 | 0.928 |
| Dry mouth | 1.38 | 1.50 | 0.88–2.08 | 1.00–2.25 | –0.12 | 0.060 |
| Heartburn | 0 | 0.15 | 0–0.38 | 0–0.65 | –0.15 | 0.045[a] |
| Decreased sexual interest | 0.73 | 1.00 | 0–1.65 | 0.23–1.92 | –0.27 | 0.082 |
| Cough | 0.77 | 1.12 | 0.27–1.15 | 0.50–1.58 | –0.35 | 0.007[a] |
| Hoarseness | 1.12 | 1.50 | 0.50–1.66 | 0.88–2.00 | –0.38 | 0.003[a] |

[a]To be tested in the confirmatory data set.

### Random data split

The data were then randomly split into an exploratory data set consisting of 307 patients (184 CHART, 123 conventional) and a confirmatory data set consisting of 308 patients (189 CHART, 119 conventional).

### Pretreatment comparisons

### *Symptoms from the RSCL*

The proportion of patients reporting moderate or severe symptoms in the exploratory data set and the confirmatory data were reasonably well balanced. Exceptions included a greater proportion of patients reporting moderate or severe symptoms in the confirmatory data set for the symptoms of worrying (24% vs 33%, $P$ = 0.013) and nervousness (14% vs 20%, $P$ = 0.047) and a greater proportion in the exploratory data set for the symptom of cough (23% vs 16%, $P$ = 0.038). Within each data set the proportion of patients reporting moderate or severe symptoms were reasonably well balanced between the treatments, although in the exploratory group there was some evidence of larger proportions in the conventional treatment group for the symptoms of hoarseness (31% vs 42%, $P$ = 0.047), cough (18% vs 31%, $P$ = 0.010), despondent feelings (11% vs 20%, $P$ = 0.038) and shortness of breath (10% vs 19%, $P$ = 0.030). In the confirmatory data set there was some evidence of a greater proportion of patients reporting moderate or severe symptoms in the conventional treatment group for the symptom of despondent feelings (12% vs 21%, $P$ = 0.040).

### *Anxiety and depression from HADS*

There was no evidence of a difference in the proportion of patients with borderline or case anxiety, or borderline or case depression, before the start of treatment in the exploratory data set when compared to the confirmatory data set. Within each exploratory and confirmatory data set, there was no evidence of a difference between the treatments.

### Exploratory data set

### *Subject-specific analysis*

***Short-term symptoms from the RSCL.*** For all but one of the 33 symptoms, analyses were based on at least 274 patients, the exception being the symptom decreased sexual interest which was based on 209 patients.

A summary of the results for all 33 symptoms from the RSCL are presented in Table 2. They are ordered by the magnitude of difference between the median SAUC score for CHART and conventionally treated patients. At the top of the Table appear the symptoms where the patients treated with CHART have a higher

*© 1999 Cancer Research Campaign*

SAUC score than conventionally treated patients. At the bottom of the Table appear the symptoms where conventionally treated patients have the higher SAUC score. The higher the score the worse the symptom is over the initial 3 months. For each symptom the median score by treatment is given together with its interquartile range and the *P*-value from the Mann–Whitney test.

For the majority of symptoms, there was no good evidence of a large difference between the two treatments over the initial 3 months. However, there were four symptoms where there was an apparent difference which therefore generated the following treatment difference hypotheses:

1. Constipation scores are higher for patients in the CHART group
2. Heartburn scores are higher for patients in the conventional group
3. Cough scores are higher for patients in the conventional group
4. Hoarseness scores are higher for patients in the conventional group.

***Short-term anxiety and depression from HADS.*** There were 271 patients (159 CHART, 112 conventional) in the analysis of anxiety. The median SAUC score was 0 for patients both on CHART and on conventional treatment, with the interquartile ranges being 0–0.38 and 0–0.35 respectively. There was no good evidence of a difference in anxiety for patients in the two treatment arms over the first 3 months ($P = 0.784$).

There were 273 patients (160 CHART, 113 conventional) in the analysis of depression. The median SAUC score was 0 for patients both on CHART and on conventional radiotherapy, with the interquartile ranges being 0–0.35 and 0–0.27 respectively. There was no good evidence of a difference in depression for patients in the two treatment arms over the first 3 months ($P = 0.214$).

### *Group-based analysis*

***Short-term symptoms from the RSCL.*** Profiles of the proportion of patients reporting ‘moderately’ or ‘very much’ for those symptoms exhibiting a difference of the order of 15% between treatment groups at any given time point are shown in Figure 2. Note that since the proportions at each assessment were not based on the same patients, consecutive data points should strictly not be connected in each of the plots. Numbers of patients contributing at each timepoint are given underneath each plot. These generated the following treatment difference hypotheses:

1. Three symptoms were reported as ‘moderately’ or ‘very much’ by more patients in the CHART group at day 21 compared to the conventional group. These symptoms and percentages were: the symptom tiredness, 55% of CHART patients and 35% of conventionally treated patients; the symptom pain, 53% of CHART patients and 34% of conventionally treated patients; the symptom lack of energy, 45% of CHART patients and 29% of conventionally treated patients. Comparing the figures at day 21 using the $\chi^2$ test gave $\chi^2 = 10.64$ on 1 df for tiredness ($P = 0.001$), $\chi^2 = 9.995$ on 1 df for pain ($P = 0.002$), and $\chi^2 = 7.399$ on 1 df for lack of energy ($P = 0.007$).
2. Two symptoms were reported as ‘moderately’ or ‘very much’ by more patients in the conventional radiotherapy group at 6 weeks compared to the CHART group. These were: the symptom cough, 35% of conventionally treated patients and 16% of CHART treated patients; the symptom hoarseness, 55% of conventionally treated patients and 28% of CHART treated patients. Comparing the figures at 6 weeks using the $\chi^2$ test gave $\chi^2 = 13.289$ on 1 df for cough ($P < 0.001$), and $\chi^2 = 20.799$ on 1 df for hoarseness ($P < 0.001$).

***Short-term anxiety and depression from HADS.*** Anxiety and depression levels did not change greatly in the short-term from those observed at the pre-treatment assessment, remaining at around 20–25% for each group, and there was no clear evidence of a large difference between treatments at any time.

### Confirmatory data set

In the confirmatory data set, analyses were only performed for those symptoms where a hypothesis had been generated from the exploratory data set.

### *Subject-specific analysis*

***Unconfirmed hypotheses.*** There is no evidence to confirm that: scores for constipation were higher for CHART patients ($P = 0.918$); scores for heartburn were higher for conventionally treated patients ($P = 0.815$); scores for cough were higher for conventionally treated patients ($P = 0.864$), or that scores for hoarseness were higher for conventionally treated patients ($P = 0.541$).

### *Group-based analysis*

***Confirmed hypotheses.*** Pain: There is evidence to confirm that this symptom was worse for patients on CHART at day 21 compared to patients on conventional radiotherapy ($P < 0.0001$). 63% of patients on CHART reported ‘moderately’ or ‘very much’ at day 21, with 39% for conventional radiotherapy (Figure 3).

*Cough:* There is evidence to confirm that this symptom was worse for patients in the conventional radiotherapy group at 6 weeks compared to the CHART group ($P = 0.006$). Thirteen per cent of patients on CHART reported ‘moderately’ or ‘very much’ at 6 weeks, with 26% for conventional radiotherapy.

*Hoarseness:* There is evidence to confirm that this symptom was worse for patients in the conventional radiotherapy group at 6 weeks compared to the CHART group ($P < 0.001$). 32% of patients on CHART reported ‘moderately’ or ‘very much’ at 6 weeks, with 53% for conventional radiotherapy.

***Unconfirmed hypotheses.*** There is no evidence to confirm that tiredness ($P = 0.398$) or lack of energy ($P = 0.105$) were worse for CHART patients at day 21.

### Long-term QOL

A total of 467 patients survived 1 year or more, of whom 367 (79%) completed their questionnaire at the 1-year assessment (227 CHART, 140 conventional). Three hundred and eight patients were expected to return their questionnaires at the 2-year assessment, of which 221 (72%) were completed (140 CHART, 81 conventional).

### *Symptoms from the RSCL*

Of the patients who completed their questionnaire, 1-year data were available for 32 of the 33 items on the RSCL for at least 97%

© 1999 Cancer Research Campaign

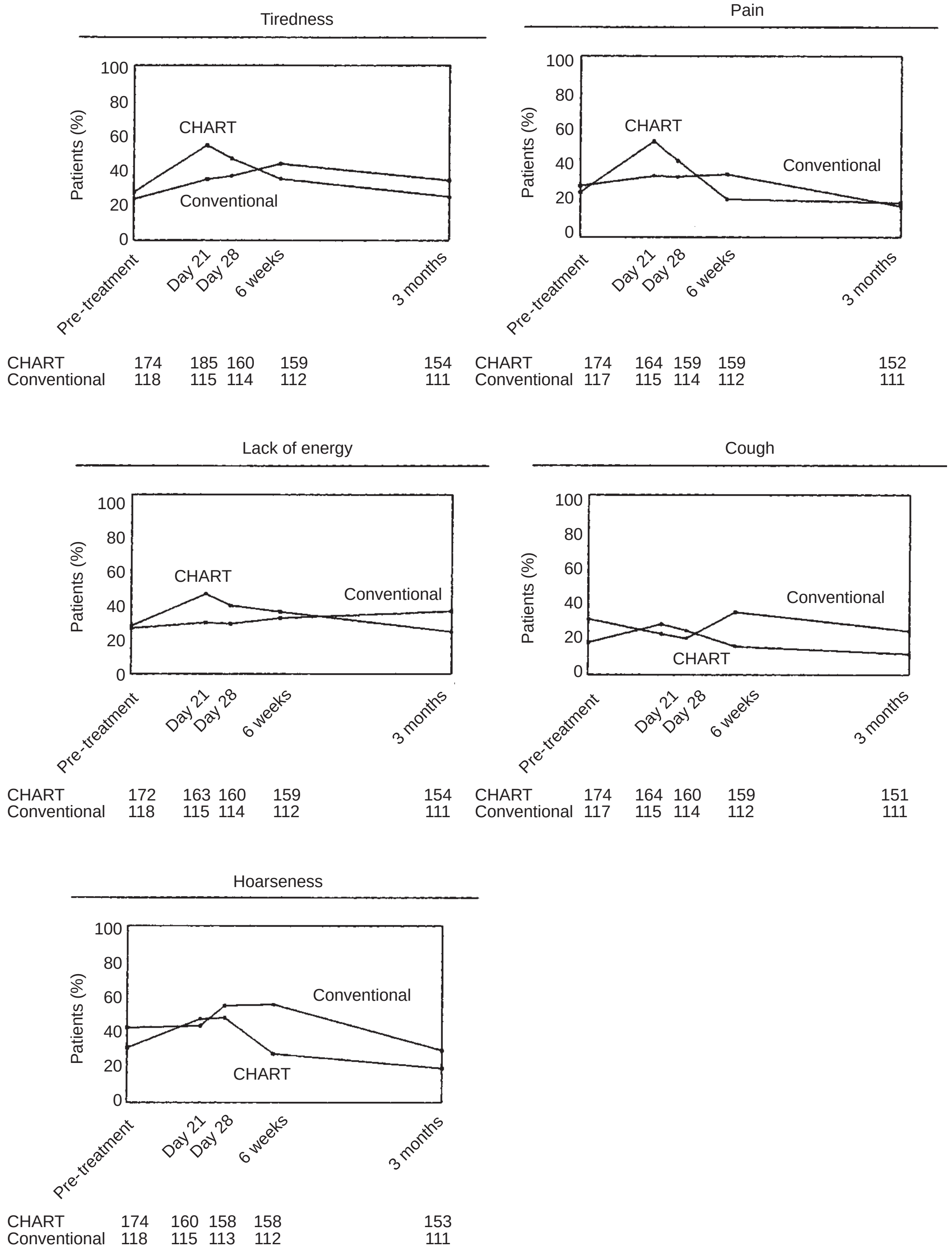

**Figure 2** Percentage of patients reporting symptoms from the RSCL as 'moderately' or 'very much' at each assessment up to 3 months, based on all available data (exploratory data set) – those symptoms where there is an apparent difference of patients (355/367), the exception being decreased sexual interest where data were available for only 77% of patients (282/367). At 2 years, data were available for 32 of the 33 items for at least 96% of patients (213/221), the exception being decreased sexual interest where data were available for 77% (170/221). The percentage of patients reporting symptoms as 'moderately' or 'very much' at 1 year and 2 years are presented in Figure 4.

At 1 year the largest and only significant difference between treatments was for decreased sexual interest, which was worse for conventionally treated patients compared to those treated with CHART (20% vs 33%, $\chi^2$ = 6.378, $P$ = 0.012). At 2 years the largest and only evidence of a difference between treatments was for sore muscles, which was worse for conventionally treated patients compared to those treated with CHART (5% vs 15%, $\chi^2$ = 6.618, $P$ = 0.010).

*© 1999 Cancer Research Campaign*

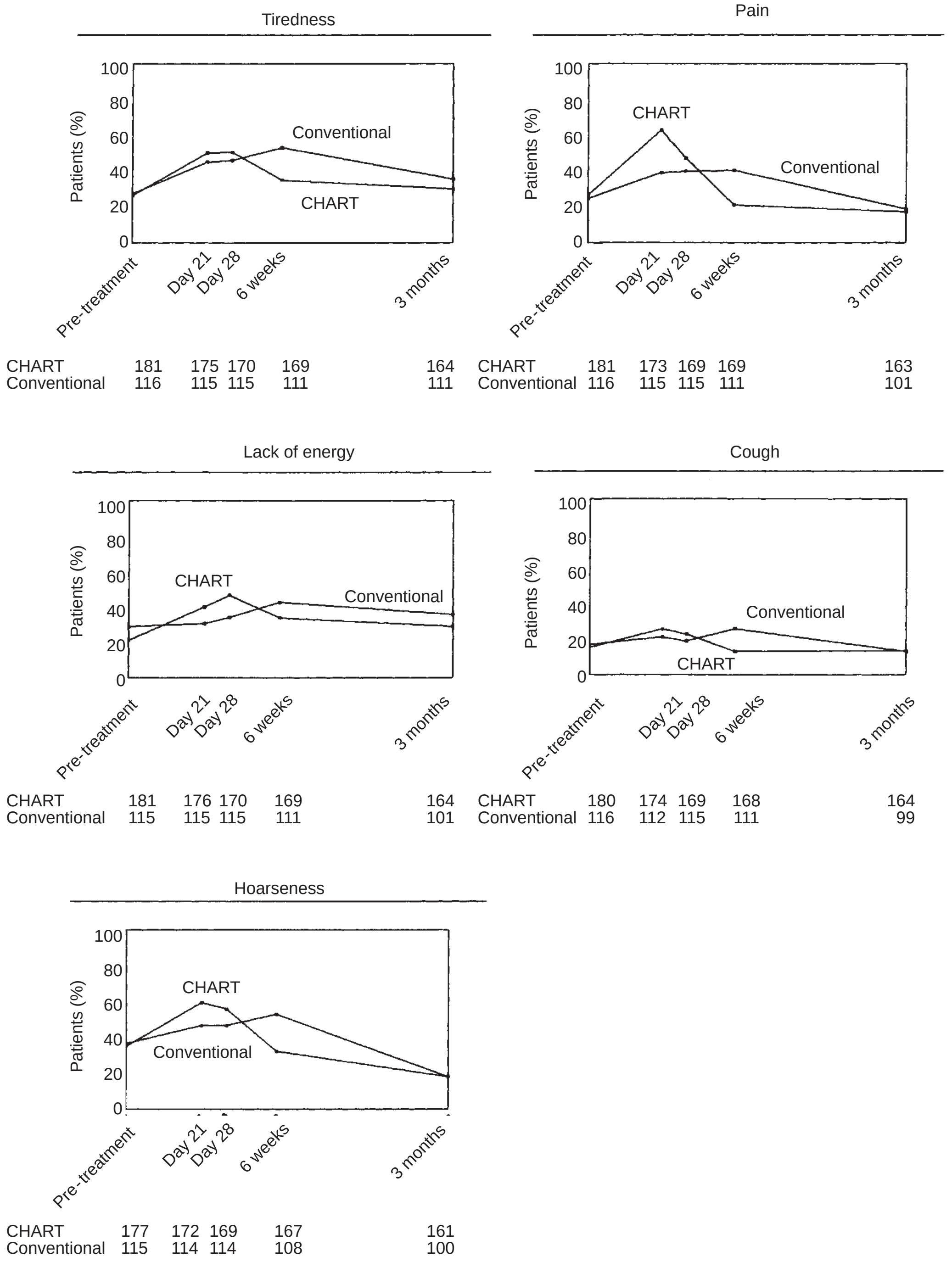

**Figure 3** Percentage of patients reporting symptoms from the RSCL as 'moderately' or 'very much' at each assessment up to 3 months, based on all available data (confirmatory data set). For symptoms in which a difference was hypothesized from the exploratory data set

However, it should be noted that with the limited number of patients with long-term data we could only reliably pick up differences of the order of 15% at 1 year (50% vs 65%, two-sided, power > 90%, $\alpha = 5\%$) and 20% at 2 years (50% vs 70%, two-sided, power = 90%, $\alpha = 5\%$).

### *Anxiety and depression from HADS*

Anxiety scores were available for 97% of patients (355/367) who completed their questionnaire at the 1-year assessment and 95% of patients (210/221) at the 2-year assessment. Depression scores were available for 97% of patients who completed their questionnaire at 1 year and 2 years (357/367 and 215/221 respectively). Anxiety and depression levels were reasonably similar to those observed at pre-treatment, and there were no large differences between treatments at 1 year or at 2 years.

### DISCUSSION

In the treatment of patients with head and neck cancer, the use of CHART resulted in no evidence of a benefit in survival compared

*© 1999 Cancer Research Campaign*

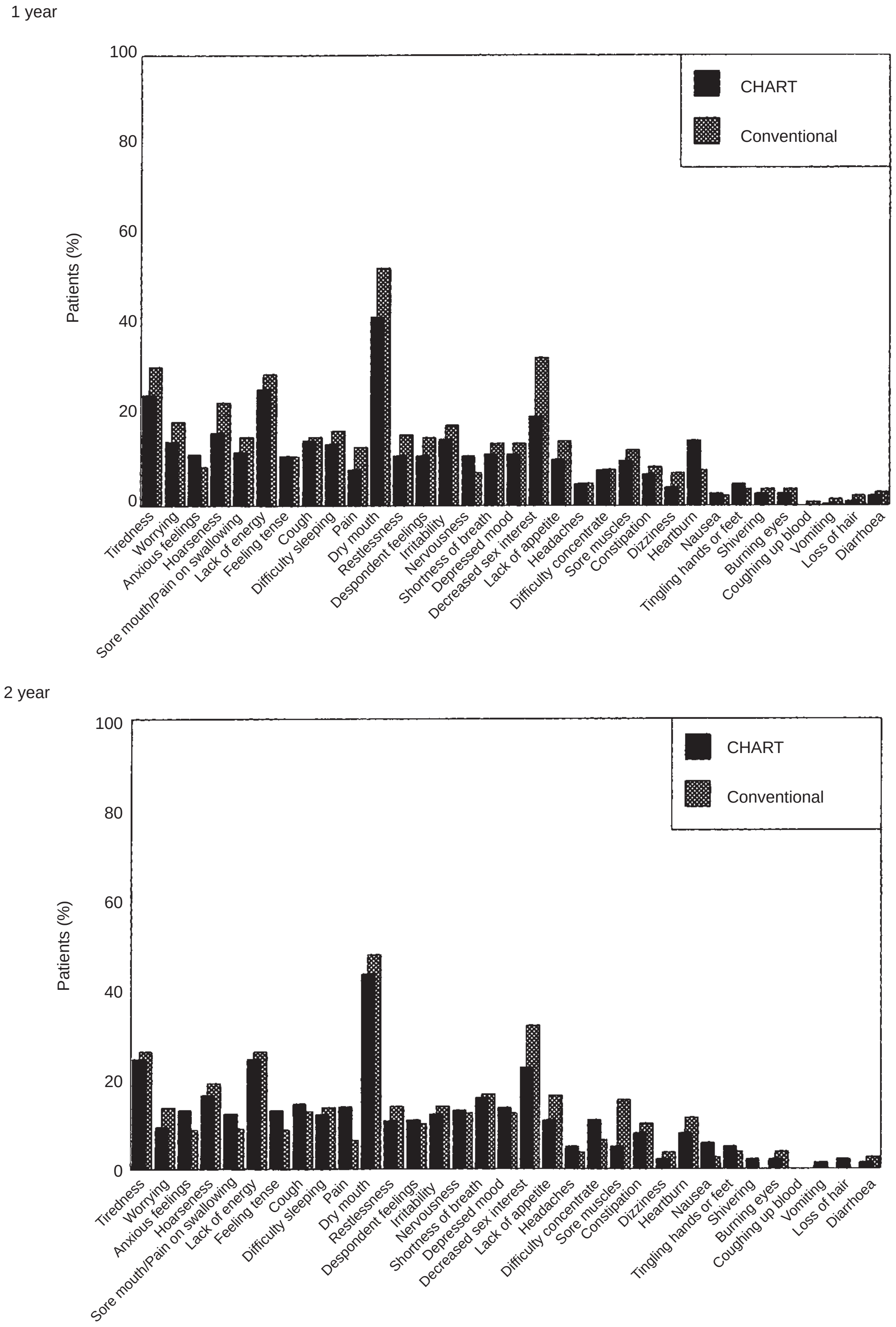

**Figure 4** Percentage of patients reporting symptoms from RSCL as ‘moderately’ or ‘very much’ in the long-term to conventional radiotherapy ($P$ = 0.62, hazard ratio = 1.05, 95% confidence interval (CI) 0.87–1.25) (Dische et al, 1997). A health economic assessment suggested that the additional cost of giving CHART is in the region of £1100 for each patient (Coyle and Drummond, 1997). Therefore the patients’ assessment of their own QOL is important in determining whether CHART offers some advantages which would be an important factor in determining the appropriate treatment for such patients.

In summary, our analyses have shown evidence of significantly worse symptoms of pain at day 21 in those treated with CHART and significantly worse symptoms of cough and hoarseness at 6 weeks in those treated conventionally. There was also evidence to suggest a greater reporting of decreased sexual interest at 1 year and sore muscles at 2 years in those treated conventionally; however, the analysis at these long-term time points were exploratory in nature and therefore firm conclusions cannot be

*© 1999 Cancer Research Campaign*

reached from these observed differences. These differences at day 21 and 6 weeks are from single time point analyses, the SAUC analyses suggest that these differences at single time points are diluted over the 3 months such that over this 3-month period there was no clear evidence of poorer or better symptoms on CHART.

There are no standard methods in analysing QOL, and probably the most appropriate recommendation is to analyse the data in several ways, and only be confident if the results are consistent (Hopwood et al, 1994). The group-based approach highlights any differences in treatments which may be occurring in the patient group as a whole at defined points in time. Although useful, such an approach does not allow for the large degree of variability between patients over time, nor does it necessarily reflect the changing patterns of symptoms over time. To allow for this in the analysis a subject-specific approach is also used.

It is interesting how in the subject-specific analysis, after randomly splitting the data into two data sets, significant differences were found for four symptoms (constipation, cough, hoarseness and heartburn) between the two arms in the exploratory data set, which were not confirmed in the confirmatory data set. This strengthens the reasoning for splitting the data into two sets, otherwise multiple comparisons may have resulted in an inappropriate interpretation. The method we used here of one data set to generate hypotheses and one to confirm them, means that we have a greater degree of confidence in any differences found.

In our analyses we found significant differences in anxiety between sexes at the pre-treatment assessment ($P < 0.001$) with 24% of males presenting with borderline or case anxiety compared with 43% of females. The NSCLC paper also found a significant difference with 14% of males presenting borderline or case anxiety compared with 45% of females ($P < 0.0001$). These results and proportions are consistent with those with anxiety disorder expected in the general population (Murphy et al, 1988). There was a non-significant difference in depression between sexes ($P = 0.095$).

In the parallel NSCLC trial the group-based analysis of the short-term data resulted in more severe sore mouth or pain on swallowing and heartburn at day 21 for those treated with CHART. This analysis of the head and neck trial reported worse symptoms for CHART at day 21, which may reflect the fact that CHART treatment had recently finished whereas the conventional group were just halfway through their course. This effect at the end of a treatment period is supported by the finding that there were worse symptoms of cough and hoarseness in the conventionally treated patients at 6 weeks, when that course of treatment ends. Both trials were similar in that the subject-specific analysis did not imply any differences in symptoms over the first 3 months. Long-term analyses in both trials were only exploratory in nature but did suggest worse symptoms of pain (NSCLC trial), decreased sexual interest and sore muscles (head and neck trial) in the conventionally treated patients. Both trials found no evidence of any differences between the CHART and conventional treatments in terms of anxiety and depression.

In our analyses we found evidence of worse symptoms of cough and hoarseness at 6 weeks in the conventionally treated patients. These two symptoms are often related and one may speculate that these symptoms are more likely to be disease related rather than treatment related. However, we must emphasize that this analysis includes both causes of such symptoms as we do not try to distinguish between them.

The overall conclusion of the analysis of these data from the head and neck trial is that in the short-term there was no evidence of any differences in psychological symptoms between conventionally treated patients and those treated with CHART. In addition, there was found to be no evidence of a difference in the physical symptoms between conventionally treated patients and those treated with CHART except for worse symptoms of pain at day 21 for those on CHART and worse symptoms of cough and hoarseness at 6 weeks for those treated conventionally. These differences subsided by 3 months.

The physical symptoms in this study correspond reasonably well with what might have been predicted from the peak acute reactions, illness and tumour response rate as reported on the clinical forms, being somewhat increased, but in shorter duration, in the CHART group. The symptoms of anxiety and depression did not differ in the two treatment groups, suggesting that they may be related to underlying disease rather than therapy. The analysis of patient reported symptoms does not give clear indication that one regimen is superior to the other in terms of ‘quality of life’, with the more severe reaction in the CHART group being offset by the longer duration of symptoms in the conventionally treated group. The QOL data seem to closely follow the information collected by the clinicians on the clinical forms. This raises the question as to how far such detailed QOL studies provide information of additional benefit in deciding optimal treatment. Only further experience and analysis in other trials will reveal whether this is a common finding.

### ACKNOWLEDGEMENT

Professor Saunders is supported by a CRC Grant, No: SP1989/0203.

### REFERENCES

Saunders M, Dische S, Barrett A, Harvey A, Gibson D and Parmar MKB on behalf of the CHART Steering Committee (1997) Continuous hyperfractionated accelerated radiotherapy (CHART) versus conventional radiotherapy in non-small cell lung cancer: a randomised multicentre trial. *Lancet* **350**: 161–165

Dische S, Saunders M, Barrett A, Harvey A, Gibson D and Parmar M on behalf of the CHART Steering Committee (1997) A randomised multicentre trial of CHART versus conventional radiotherapy in head and neck cancer. *Radiother Oncol* **44**: 123–136

De Haes JCJM, Van Knippenberg FCE and Neijt JP (1990) Measuring psychological and physical distress in cancer patients: structure and application of the Rotterdam Symptom Checklist. *Br J Cancer* **62**: 1034–1038

Zigmond AS and Snaith RP (1983) The Hospital Anxiety and Depression Scale. *Acta Psychiatr Scand* **67**: 361–370

Bailey AJ, Parmar MKB and Stephens RJ (1997) Quality of life (QoL) in the CHART randomised trial in non-small cell lung cancer (NSCLC): Short and long term patient reported symptoms. *World Lung Conference* 205 (Abstract 794)

Bailey AJ, Parmar MKB and Stephens RJ (1998) Patient-reported short-term and long-term physical and psychological symptoms: Results of the CHART randomised trial in non-small cell lung cancer. *J Clin Oncol* **16**: 3082–3093

Cox DR, Fitzpatrick R, Fletcher AE, Gore SM, Spiegelhalter DJ and Jones DR (1992) Quality-of-Life assessment: can we keep it simple? (with discussion). *J R Statist Soc* A, **155**: 353–393

COMPACT Steering Committee (1991) Improving the quality in clinical trials in cancer. *Br J Cancer* **63**: 412–415

SAS (1989) SAS Institute Inc., Cary, North Carolina.

Altman DG (1991) *Practical Statistics for Medical Research.* Chapman & Hall: London

Coyle D and Drummond MF on behalf of the CHART steering committee (1997) Costs of conventional radical radiotherapy versus continuous hyperfractionated

 © 1999 Cancer Research Campaign accelerated radiotherapy (CHART) in the treatment of patients with head and neck cancer or carcinoma of the bronchus. *Clin Oncol* **9**: 313–321

Hopwood P, Stephens RJ and Machin D (1994) Approaches to the analysis of quality of life data: experiences gained from a Medical Research Council Lung Cancer Working Party palliative chemotherapy trial. *Quality of Life Res* **3**: 339–352

Murphy JM, Olivier DC, Monson RR, Sobol AM and Leighton AH (1988) Incidence of depression and anxiety: The Stirling County Study. *Am J Public Health* **78**: 534–540

*© 1999 Cancer Research Campaign* 
